# An Unusual Cause of Recurrent Urinary Retention in an Adolescent Female

**DOI:** 10.7759/cureus.5136

**Published:** 2019-07-15

**Authors:** Induja Gajendran, Omosede Uzamere, Kaitlyn McSurdy, Adebayo Adeyinka, Louisdon Pierre

**Affiliations:** 1 Pediatrics, The Brooklyn Hospital Center, New York, USA; 2 Pediatrics, The Brookyln Hospital Center, New York, USA; 3 Pediatrics, St. George's University, Grenada, GRD

**Keywords:** uterus didelphys, urinary retention, renal agenisis, herlyn werner wunderlich syndrome, adolescent, abdominal pain

## Abstract

Herlyn-Werner-Wunderlich syndrome (HWWS) is a rare, combined Mullerian and Mesonephric duct anomaly characterized by the triad of uterus didelphys, obstructed hemivagina and ipsilateral renal agenesis. We present the case of an otherwise healthy 16-year-old female with acute urinary retention secondary to HWWS. The diagnosis was established with abdominal ultrasound and Magnetic Resonance Imaging (MRI). The patient subsequently underwent surgical resection of the vaginal septum resulting in relief of obstruction. Clinical symptoms in patients with HWWS typically present after menarche with progressive hematometra causing pain and compression of localized structures. Even though ultrasound can help in the diagnosis, MRI is the best choice of imaging for the visualization of these anomalies. The diagnosis of HWWS is important to consider in young females of reproductive age presenting with symptoms of obstruction of adjacent structures. Our patient presented with acute urinary retention which is a rare symptom in this entity. A high index of clinical suspicion and awareness of the syndrome are required to make a speedy diagnosis and prevent future complications.

## Introduction

Herlyn-Werner-Wunderlich syndrome (HWWS) also known as OHVIRA (Obstructed Hemivagina and Ipsilateral Renal Anomaly) is a rare, combined Mullerian and Mesonephric duct anomaly characterized by the triad of uterus didelphys, obstructed hemivagina and ipsilateral renal agenesis [1]. Its estimated occurrence is between 0.1% and 3.8% [2]. It is hypothesized that HWWS is caused by the anomalous development of Müllerian and Wolffian ducts [1]. It is generally observed in adolescents and young women within 1-2 years post-menarche [3]. Through a review of literature, some known clinical presentations that have occurred in adolescents include pelvic pain, recurrent spontaneous abortion, abdominal pain with pyocolpos, urinary tract infections, pelvic mass and dysmenorrhea [4-8]. During infancy, maternal hormone influence may produce symptoms due to the collection of secretions with outflow obstruction in the hemivagina. Older children may develop hematocolpos, hematometra or even hematosalpinx [9]. The precise etiology and pathogenesis of HWWS are still unknown [10].

## Case presentation

A 16-year-old female presented to our pediatric emergency department (ED) with complaints of worsening dysuria with bladder fullness and decreased urinary output for 4-5 hours. Associated complaints included constipation, rectal pain and feeling a lump in the rectum while straining to defecate, partially alleviated with laxative use. Our patient reported a recurrence of such symptoms for the past few months, specifically around her menstrual period, but with spontaneous resolution. She denied any history of fever, hematuria, blood in the stool, vomiting or abdominal pain. She had a history of urinary tract infection (UTI) five months prior to the presentation that resolved after antibiotics. The patient had no history of abdominal surgeries. She admits to smoking marijuana occasionally. She denied being sexually active nor any history of sexually transmitted infections. She attained menarche one year ago and has regular menstrual cycles; her last menstrual period was 14 days before presentation. 

Upon physical exam in the ED, she had fullness and mild tenderness in the suprapubic area. The rectal exam showed mild external hemorrhoids with no stool impaction. Complete blood count showed leukocytosis with neutrophilic predominance. Electrolyte panel showed initial hyponatremia, which resolved with intravenous fluids. Due to worsening pain, ketorolac was given as an analgesic, with minimal relief. Therefore, her bladder was catheterized and 800ml of urine was drained, which provided significant relief. Urinalysis did not show any signs of infection. The patient was started on ceftriaxone, nitrofurantoin and phenazopyridine due to continued symptoms of urinary retention and dysuria. After the initial drainage of urine, she was able to void by herself though with a slow stream. She was then admitted to the inpatient pediatric unit for further evaluation. 

**Figure 1:**
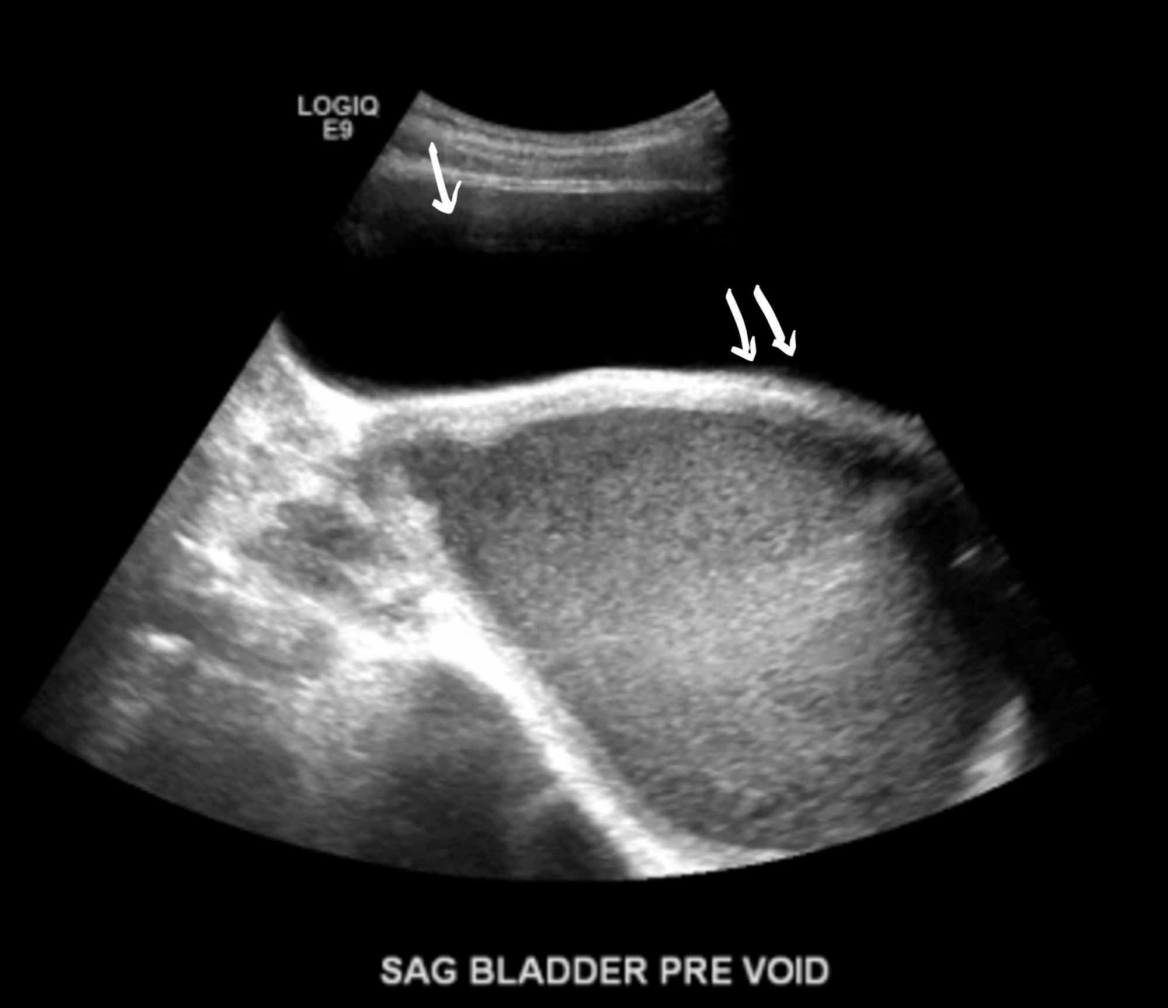
Ultrasound of bladder. Single arrow: significant postvoid urinary bladder residual volume of 264 ccs. Double arrows: well-defined structure of uncertain etiology posterior to the urinary bladder with a dominant component measuring approximately 12.2 cm.

**Figure 2:**
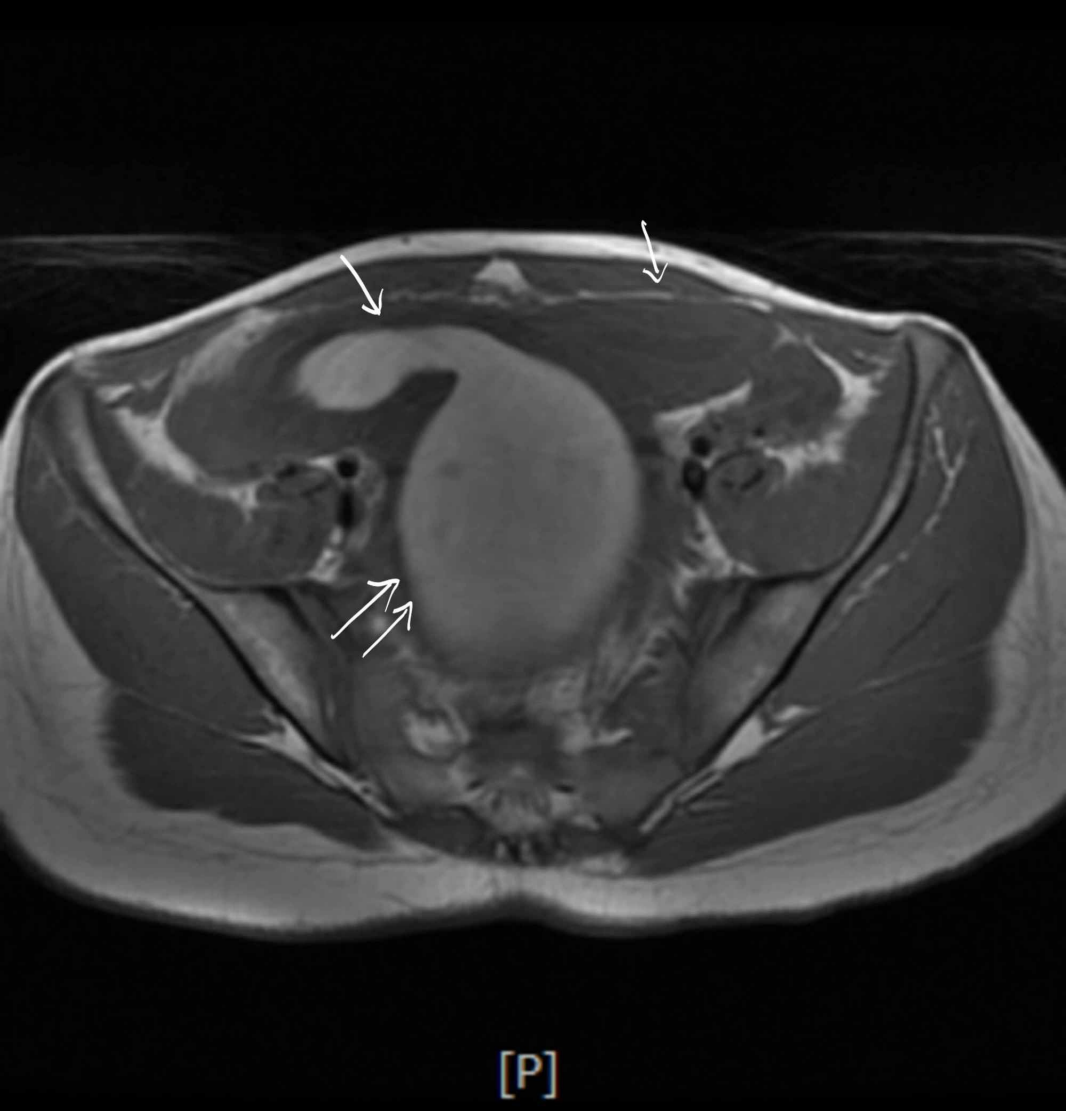
Double arrows: the uterus is enlarged, measuring 16.0 x 10.4 x 15.1 cm. Single arrow: the appearance of two horns which are widely divergent.

**Figure 3:**
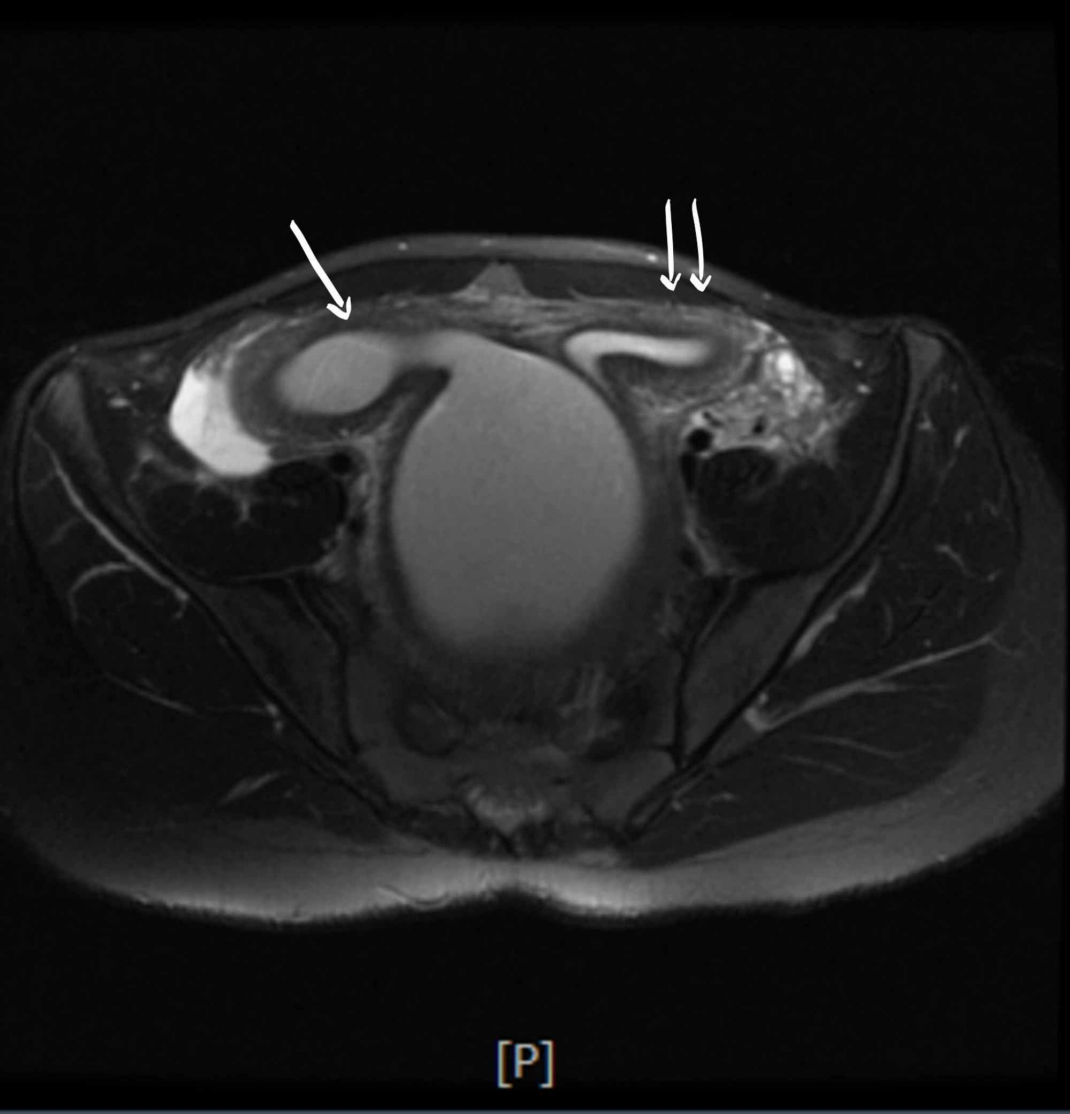
Single arrow: the endometrial cavity in the right-sided horn distended up to 2.9 cm in diameter and containing T1 hyperintense and intermediate T2 signal material suggestive of blood products. Double arrows: the endometrium in the left-sided horn measures 1.4 cm in thickness.

**Figure 4:**
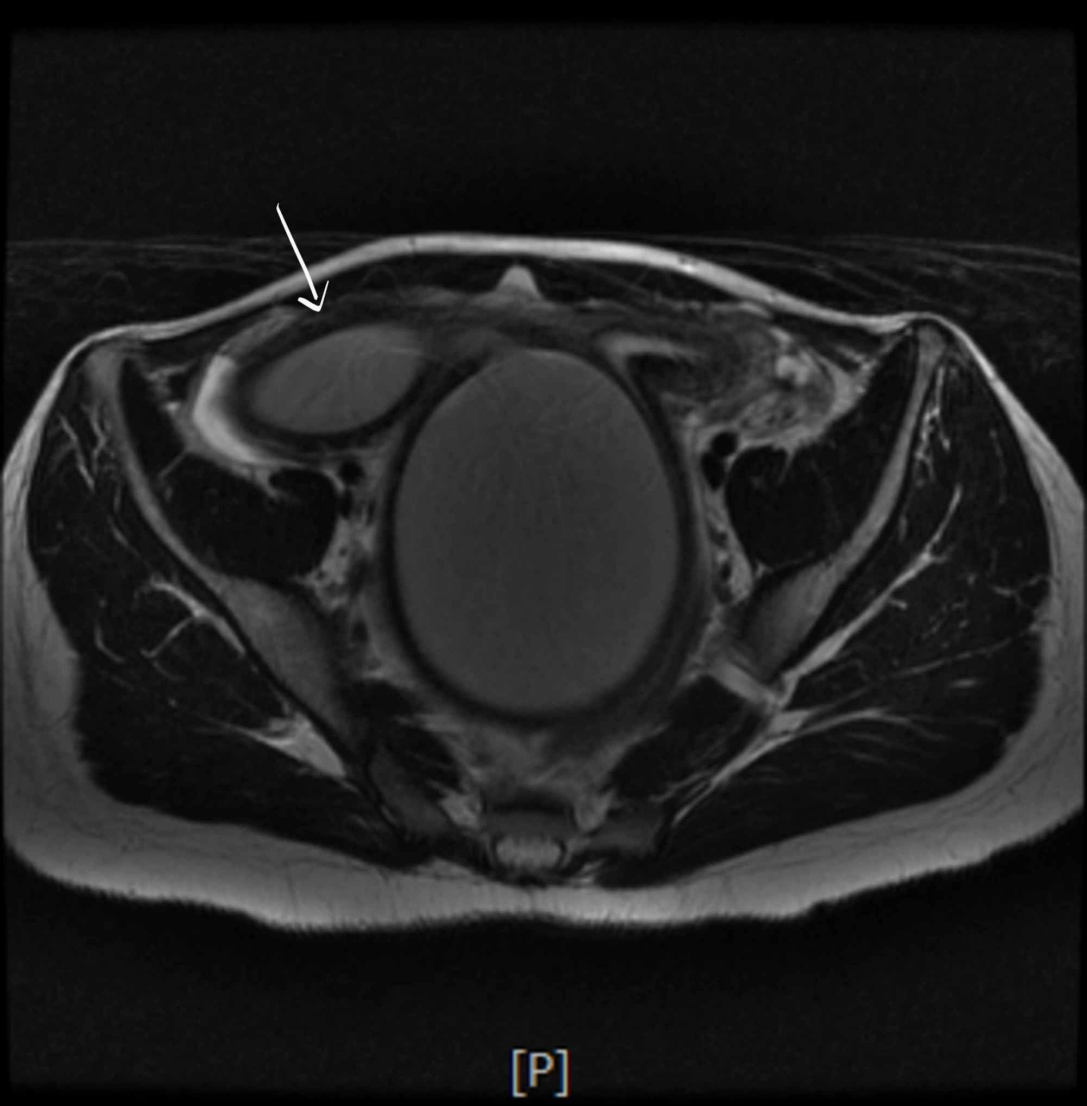
Single arrow: the right-sided horn measures 11.0 x 9.0 cm in diameter and contains products of similar signal, also likely representing blood.

**Figure 5:**
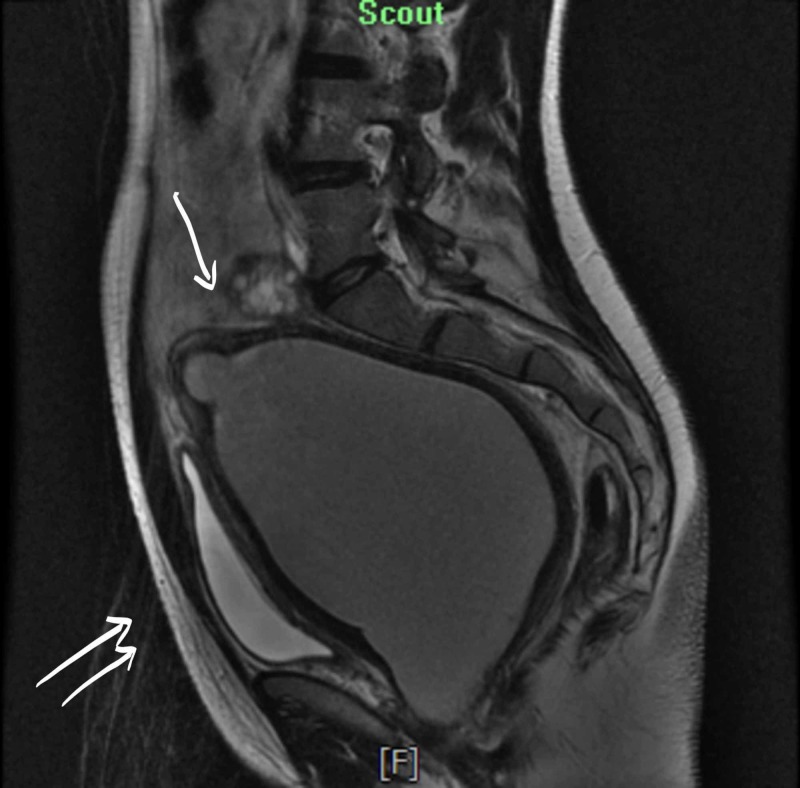
Single arrow: the right-sided horn is contiguous with a markedly distended vagina. Double arrows: the urinary bladder is compressed by the enlarged uterus however is otherwise unremarkable.

**Figure 6:**
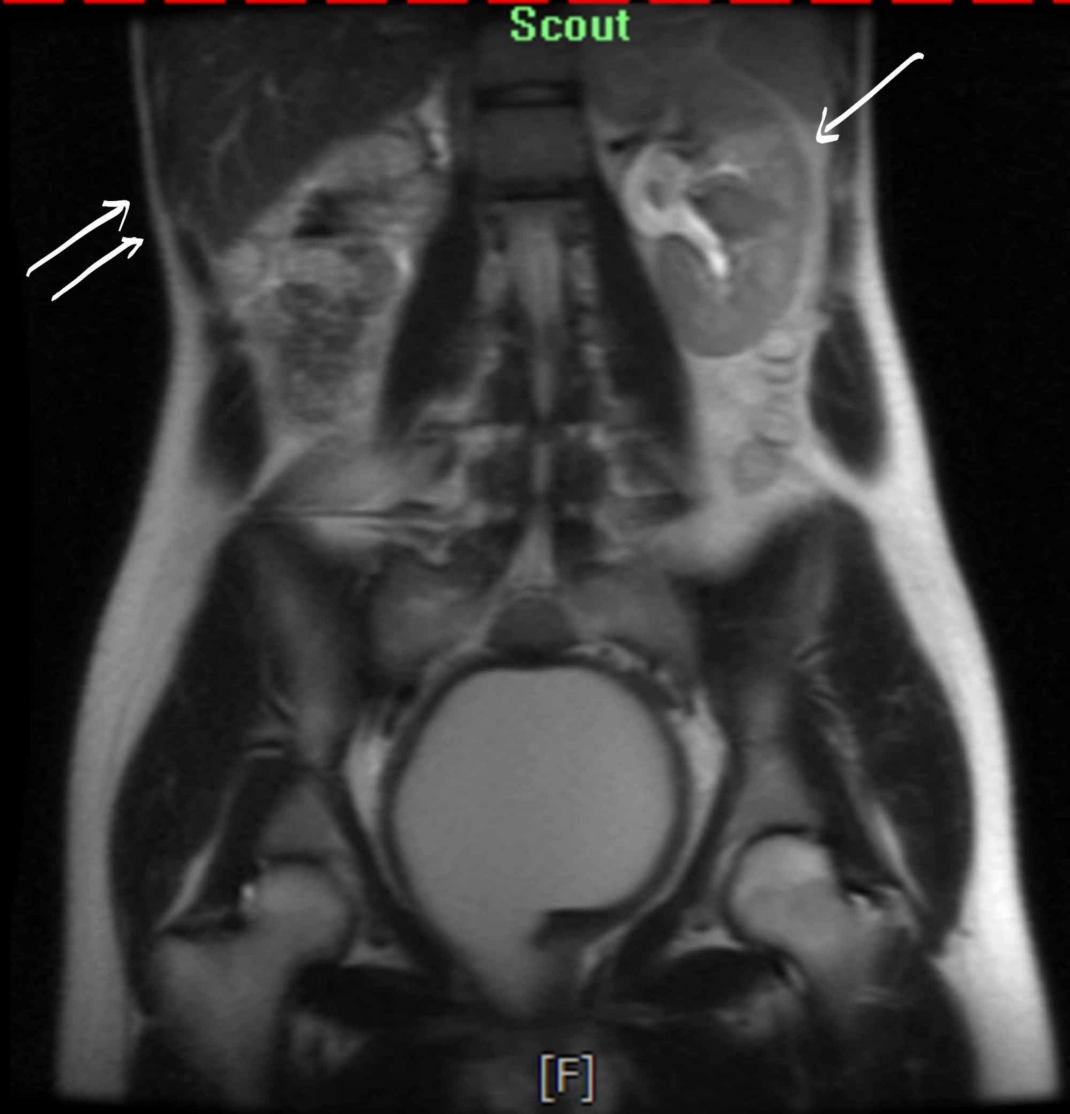
Single arrow: 3.9 cm likely simple cyst with thin septation seen in the left kidney. Double arrows: the right kidney is not visualized.

**Figure 7:**
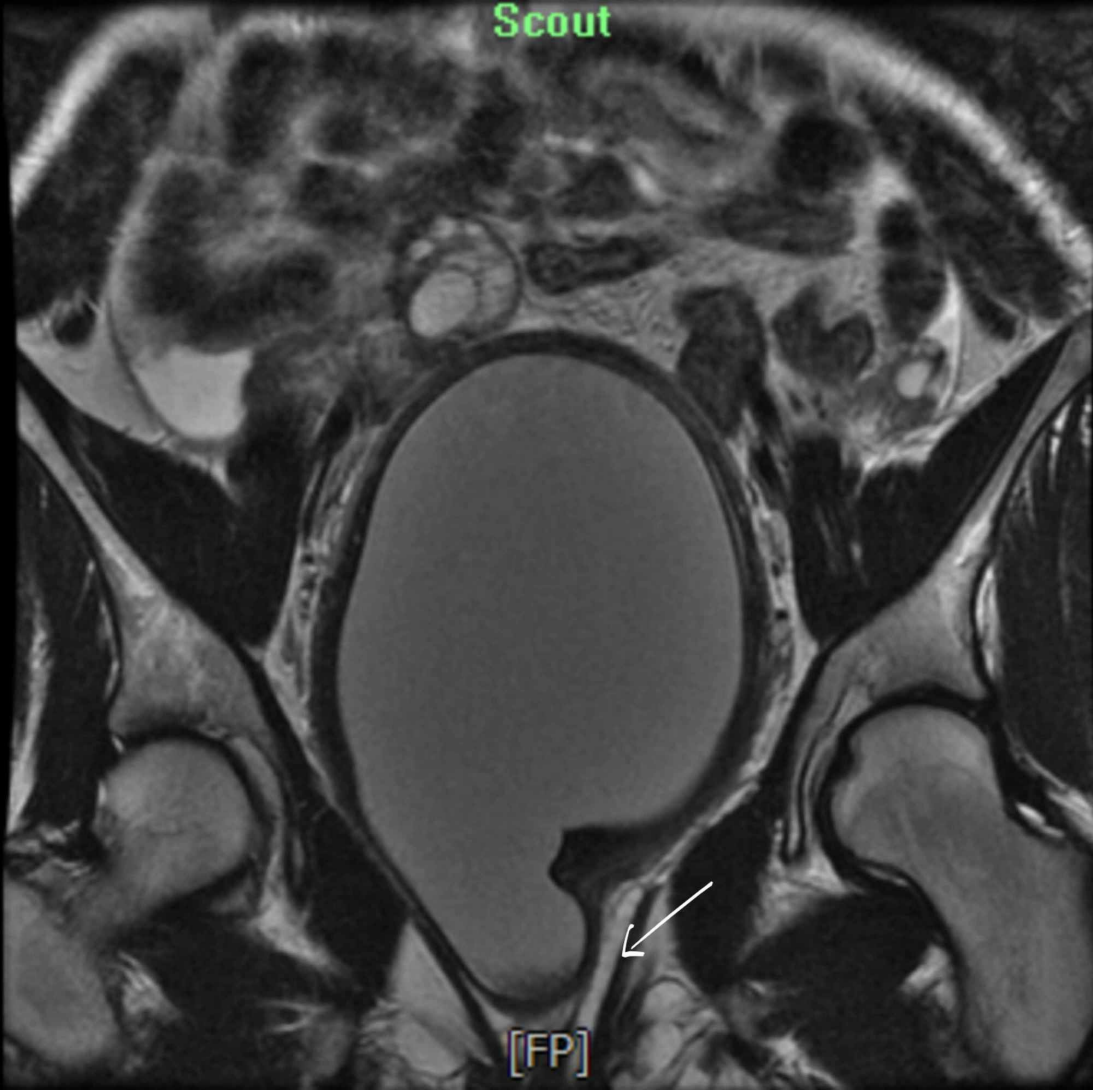
Single arrow: a left-sided upper vagina which is collapsed. The lower vagina is normal and appears to have a common channel. This appearance is suggestive of uterus didelphys with right-sided hematometrocolpos.

A few hours later she developed urinary retention again and hence had to be re-catheterized; at that time 900ml of urine was drained. Antibiotics were switched to cefepime with continued pain control and IV fluids. She continued laxatives but had a poor appetite and did not have a bowel movement since admission. She developed severe rectal pain and the rectal exam revealed a firm mass anterior to the rectal vault. No active bleeding or fecal impaction was appreciated. Bladder ultrasound (Figure 1) revealed a significant post-void urinary bladder residual volume; a well-defined structure of uncertain etiology posterior to the urinary bladder with a dominant component measuring approximately 12.2 cm. The renal sonogram revealed non-visualization of the right kidney; 2.4 cm mid-left renal cyst with partial internal septation; with no evident hydronephrosis. A follow-up MRI of the abdomen and pelvis (Figure 2 - 7) revealed signs of uterus didelphys with right-sided hematometrocolpos and right renal agenesis. Findings likely representing Herlyn-Werner-Wunderlich syndrome. The urinary bladder was decompressed with an indwelling catheter. Renal function was monitored and remained normal throughout admission. She had a negative urine culture and sexually transmitted infection panel. Urology and gynecology were consulted and as per their recommendations, bladder decompression and empiric antibiotics were continued. Finally, the patient was transferred to a higher care center for surgical correction, where she underwent surgical resection of the vaginal septum; 800ml of hematocolpos and hematometrium evacuated. The patient recovered well and was discharged home.

## Discussion

Among patients with HWWS, an initial presentation with urinary retention was found to be rare; an extensive literature review reports around 10 cases with hematocolpos causing urinary retention [5]. Most cases report a history of regular menstrual periods up until the obstruction of the hemivagina results in pelvic pain or obstruction symptoms.

A didelphic uterus is suggestive of an embryologic arrest occurring during the eighth week of gestation which ultimately affects the Mullerian and Metanephric ducts [7]. Uterus didelphys represents a failure of fusion of the paired Mullerian ducts. The obstructed hemivagina results from a transverse vaginal septum, which represents vertical fusion defect consequent to non-resorption of tissue between the vaginal plate that originates from the urogenital sinus and the caudal aspect of the paired Mullerian ducts [11]. The renal agenesis on the ipsilateral side of the obstructed hemivagina reflects the close association of the Wolffian duct relative to the Mullerian ducts in embryological development [12]. An important point to note in HWWS is that renal agenesis is ipsilateral to the dilated uterine cavity. In the case of unicornuate uteri, the renal anomalies are ipsilateral to the rudimentary or absent uterine horn [13]. Patients with this syndrome present with cyclical dysmenorrhea, which evolves into persistent pelvic pain. Nearly all of them are initially misdiagnosed clinically [9]. 

Our patient presented with recurrent urinary retention. Anatomically, the female urethra is short and straight with a large enough diameter; as a result, urinary retention is a rare occurrence in females relative to males. Urinary retention occurs in this instance as a result of haematocolpos. With the increased size and external compression, the uterus can cause urethra angulation which leads to an obstruction [14]. We also hypothesized that haematocolpos reduces the extrusion capacity of the urethral sphincter by irritating the sacral plexus [11]. Though ultrasound can be used for visualization of the genitourinary structures, MRI is the modality of choice for the diagnosis of HWWS and other such anomalies due to the better anatomic delineation of pelvic structures and higher sensitivity for blood products [15, 16].

The diagnosis of HWWS is important to consider in young females of reproductive age presenting with symptoms of obstruction of adjacent structures [17]. Generally, clinical suspicion for this condition is usually is low because: (i) the patient menstruates regularly from the non-obstructed horn; hence outflow obstruction is not a common symptom, (ii) the condition is in itself is very rare and (iii) adolescents usually present with cyclic dysmenorrhoea and are initially treated with anti-inflammatory drugs or oral contraceptives by primary physicians which may diminish menstrual flow [1]. Early diagnosis after menarche followed by excision and marsupialization of the blind hemivagina offers complete relief of symptoms and preserves reproductive potential [18]. Laparoscopic vaginal septum excision is the treatment of choice for HWWS. A successful pregnancy is achieved eventually in 87% of patients, while 23% have the risk of abortions [18]. If treatment is delayed, complications may develop, such as endometriosis caused by retrograde menstruation, infections, infertility, and pelvic adhesions [7, 17]. A high index of clinical suspicion and awareness of the syndrome are required to make a speedy diagnosis and prevent future complications [19].

## Conclusions

OHVIRA/HWW syndrome with urinary retention is a rare clinical presentation. Ultrasound and MRI findings can collectively aid in assessing the anatomy of the uterus, absence of the ipsilateral kidney and the nature of the fluid content in the obstructed hemivagina. Owing to its rarity, HWWS is usually not included in the differentials for acute urinary retention in a young woman. Since this condition can be treated by vaginal septum excision, a high index of clinical suspicion is necessary as a delay in diagnosis may worsen the associated risk of endometriosis, or adhesions from chronic infections with subsequent infertility.
